# Lung ultrasound B-lines predict adverse clinical outcomes in adults with sepsis in a resource-limited emergency department

**DOI:** 10.3389/fmed.2026.1785703

**Published:** 2026-02-19

**Authors:** Kamonwon Ienghong, Korakot Apiratwarakul, Dhanu Gaysonsiri, Lap Woon Cheung

**Affiliations:** 1 Department of Emergency Medicine, Faculty of Medicine, Khon Kaen University, Khon Kaen, Thailand; 2 Department of Pharmacology, Faculty of Medicine, Khon Kaen University, Khon Kaen, Thailand; 3 Department of Emergency Medicine, Li Ka Shing Faculty of Medicine, The University of Hong Kong, Pokfulam, Hong Kong SAR, China

**Keywords:** emergency service, health resources, sepsis, treatment outcome, ultrasonography

## Abstract

**Background:**

Sepsis increases the risk of acute respiratory distress syndrome (ARDS) and other respiratory complications. Point-of-care lung ultrasound (POCUS) detection of B-lines may facilitate early risk stratification where advanced imaging is limited. This study evaluated the association between B-lines on lung ultrasound and clinical outcomes in adult sepsis patients treated in a resource-limited emergency department.

**Methods:**

A retrospective observational study was conducted on sepsis patients treated in the emergency department from January to December 2024. Ultrasound documentation and electronic medical records were analyzed to compare overall mortality, hospital length of stay, and mechanical ventilator use between patients with and without B-lines. Primary outcomes were in-hospital mortality, mechanical ventilator use, and hospital length of stay. Descriptive statistics compared outcomes between patients with and without B-lines. Multivariable logistic regression was used to estimate the adjusted association between B-lines and hospital mortality, controlling for clinically relevant confounders.

**Results:**

Among 184 sepsis patients, 56 (30.43%) exhibited B-lines on lung ultrasound. Mortality rates (16.07% vs. 4.68%, *p* < 0.001), mechanical ventilation use (39.28% vs. 7.03%, *p* < 0.001), and median hospital stay duration (11 vs. 6 days, *p* < 0.001) were compared between the B-lines group and the non-B-lines group. In the multivariable logistic regression analysis, the B-lines group exhibited a significantly higher hospital mortality rate (adjusted odds ratio, 4.6; 95% confidence interval, 2.5–5.5) compared to the non-B-lines group.

**Conclusion:**

The presence of B-lines on lung ultrasound in sepsis patients is significantly associated with increased mortality, higher rates of mechanical ventilation, and prolonged hospital stays. These findings support the potential utility of POCUS B-line assessment for early risk stratification in resource-limited emergency settings.

## 1 Introduction

Sepsis is a prevalent condition in the emergency department (ED), with a global incidence of 12–15% and a case fatality rate of 16–20% (1–3). More than one-third of sepsis patients develop acute respiratory distress syndrome (ARDS), which is typically more severe than sepsis without ARDS, leading to poorer prognoses and higher mortality rates (2–5). Sepsis induced ARDS contributes significantly to critical care mortality worldwide, with rates exceeding 40% in resource-limited settings despite advances in management protocols.

ARDS is diagnosed based on the Berlin definition (6), which includes the onset of respiratory symptoms, chest imaging, and oxygenation levels. Chest radiographs have suboptimal interobserver reliability and are often under-recognized in clinical settings, while computed tomography (CT) scans are not widely available. Moreover, no biomarker for ARDS is currently endorsed in clinical practice (7). Recent advancements in ARDS diagnosis (7) have resulted in a new global definition that, for the first time, formally integrates lung ultrasound findings as diagnostic criteria, especially in resource-constrained environments where chest radiography or CT may be inaccessible or impractical. This global definition acknowledges particular ultrasound patterns such as bilateral B-line predominance, with or without consolidations and pleural abnormalities as acceptable imaging criteria for diagnosing ARDS when conventional radiographic imaging is unavailable. This development recognizes the increasing body of evidence supporting the accuracy of lung ultrasound and its specific applicability in low- and middle-income nations.

Point-of-care lung ultrasound (LUS) has developed into a swift, non-invasive, and portable bedside instrument for identifying pulmonary pathology. B-lines, which are vertical hyperechoic artifacts originating from the pleural line, signify elevated extravascular lung water and interstitial involvement. B-lines are a sensitive but nonspecific finding that could result from various etiologies, including community-acquired pneumonia, cardiogenic pulmonary edema, iatrogenic fluid overload due to aggressive resuscitation, chronic interstitial lung diseases, and acute respiratory distress syndrome (ARDS). In the emergency department, excessive fluid administration during initial resuscitation may frequently lead to the development of B-lines. Although there is no specificity for an individual diagnosis, the identification of B-lines in septic patients may yield significant prognostic implications. The presence of pulmonary pathology indicated by B-lines may identify patients necessitating closer monitoring, earlier respiratory support, or adjustments in fluid management strategies, independent of the underlying etiology. In resource-constrained environments lacking advanced imaging techniques and limited access to invasive monitoring, LUS serves as an effective alternative for bedside evaluation (8–10).

The prognostic value of B-lines identified through point-of-care lung ultrasound in septic patients arriving at the emergency department remains inadequately defined, especially in settings with limited resources. Determining the correlation between B-line presence and adverse outcomes may assist clinicians in risk stratification and inform management decisions in the absence of advanced diagnostic resources. This study aimed to assess the association between B-lines identified through point-of-care lung ultrasound and clinical outcomes in adult patients with sepsis presenting to the emergency department in a resource-constrained environment.

## 2 Materials and methods

### 2.1 Study design and setting

This retrospective study analyzed computerized medical records from January to December 2024 at the ED of Srinagarind Hospital. As the leading tertiary care medical center in Northeastern Thailand, Srinagarind Hospital operates within the Faculty of Medicine at Khon Kaen University and serves 60,000 to 70,000 emergency patients annually. The primary objective was to evaluate the recognition of B-lines via lung POCUS by emergency physicians and its impact on clinical outcomes, including mortality rates, mechanical ventilation use, and hospital length of stay in adult sepsis patients in the emergency department.

### 2.2 Participants

Patients aged 18 years or older who were diagnosed with sepsis based on the Sepsis-3 criteria (11) and admitted to the ED during the study period were included. Patients were excluded if they had a history of heart failure classified as New York Heart Association class III–IV, end-stage chronic kidney disease, chronic liver disease (Child-Pugh score > 10), interstitial lung disease, active cancer, chest trauma or a chest tube at admission, acute myocardial infarction, cardiogenic shock, were transferred from another hospital, missing data or lacked ultrasound documentation.

### 2.3 Data gathering

The principal investigator retrieved electronic medical records using the Health Object Program®, an accredited electronic medical records system, and assigned an anonymous identifier to each patient. Collected data included demographic information [age, sex, triage level according to the Emergency Severity Index triage system (12), past medical history, etiology of sepsis, fluid balance at admission, diuretic used, renal replacement therapy, in-hospital mortality, hospital length of stay, and mechanical ventilation usage].

Sepsis was defined based on the Sepsis-3 criteria, which require a suspected infection accompanied by organ dysfunction, as evidenced by an increase in the Sequential Organ Failure Assessment (SOFA) score of 2 points or more. This study employed the quick SOFA (qSOFA) score of ≥2, which encompasses a respiratory rate of ≥22 breaths per minute, altered mental status, or a systolic blood pressure of ≤100 mm Hg, as a bedside screening instrument in the emergency department to identify patients with suspected infections who are likely experiencing organ dysfunction, in accordance with Sepsis-3 guidelines for situations where complete SOFA scores are not readily accessible.

ARDS was characterized by acute hypoxemia, defined as a partial pressure of arterial oxygen to the fractional concentration of inspired oxygen ratio of ≤300, along with bilateral pulmonary infiltrates on chest radiographs that were not solely attributable to congestive heart failure or fluid overload (13).

POCUS was routinely performed within 2 h of ED arrival for patients with suspected sepsis, including ultrasound evaluations of the heart, lungs, and inferior vena cava. Each patient was assessed by an attending physician, including emergency medicine residents who had completed at least 1 month of ultrasound training and emergency physicians with over 5 years of POCUS experience. However, the decision to perform POCUS was at the discretion of the attending physician. This study consistently followed the lung ultrasound approach outlined in the BLUE protocol (14). B-line artifacts were evaluated through visual examination by physicians, with more than three B-lines considered an abnormal finding. Ultrasound findings were documented in the medical records. POCUS was performed using the Mindray M9 (Mindray, Shenzhen, China) to obtain the ultrasound recording. The ultrasound probes used for detecting B-lines included the Mindray C5-1s curved array transducer, operating within a frequency range of 1.0 MHz to 5.0 MHz under a lung software preset. Ultrasound video recordings were stored for at least 6 s per clip, in accordance with hospital protocols. The videos and ultrasound images remained on the device, from which investigators retrieved the data using a Universal Serial Bus drive within 72 h of acquisition. Monthly integrity assessments were conducted on all stored files to ensure regular backup validation. All file access was recorded with timestamps and user identification for access logging purposes.

Patients with documented B-lines on lung ultrasound were subsequently evaluated by the principal investigator, who verified the diagnosis of ARDS based on established criteria. The management of sepsis patients followed standard practices determined by attending physicians. Resuscitation and treatment decisions for each patient were made by the clinical team responsible for their care in the ED.

Data was collected and systematically organized into a research database. All ultrasound recordings were stripped of patient identifiers, clinical information, and timestamps before review. Images were assigned random study numbers unlinked to patient outcomes or clinical data. Ultrasound interpretations were subjected to a blind evaluation by two independent emergency physicians possessing advanced POCUS certification, conducted without access to patient clinical outcomes, laboratory results, or treatment interventions administered. Reviewers were provided with standardized instructions that included uniform B-line criteria, quantitative threshold (>3 B-lines deemed abnormal), and standardized evaluation forms for documentation. Two independent investigators reviewed the data, correcting any redundant entries. In cases of data inconsistencies, the senior investigator, with over a decade of experience in the field, was consulted to ensure data accuracy.

### 2.4 Statistical analysis

We formulated a design-appropriate rationale for our multivariable prognostic analyses by focusing requirements on events-per-variable (EPV) for logistic regression. We decided on 5 EPV as the principal criterion, in accordance with widely referenced recommendations for concise prognostic models (15, 16). The necessary sample size was determined based on expected event rates: Initially, in-hospital mortality is predicated on an event proportion of 12% (15). With a minimum of 5 EPV, E = 30, the requisite sample size was 250. Regarding mechanical ventilation, the assumed event proportion is 25. The required sample size was 120, based on a minimum of 5 EPV. Based on retrospective data from an all-eligible cohort over a fixed 12-month period, the requisite sample size for event outcomes was a minimum of 120 patients.

Continuous variables were summarized using the median and interquartile range, while categorical variables were summarized using counts and percentages. To compare overall mortality, hospital length of stay, and mechanical ventilator utilization between patients with and without B-lines, a *t*-test or median test, chi-square test, and univariate/multivariate logistic regression models were used.

A multivariate logistic regression model was developed using two distinct models: one for mechanical ventilation use and one for in-hospital mortality. Primary predictor was presence of B-lines on lung ultrasound. We used purposeful selection; age, sex, PaO2/FiO2, and sepsis etiology were retained *a priori*. Additional covariates (triage level, comorbidities, fluid balance at admission, diuretic use, renal replacement therapy, vasopressor use) with univariable *p* < 0.20 were entered and retained if statistically significant or if their removal changed the B-lines coefficient by ≥10%. Data were entered into Microsoft Excel and analyzed using IBM SPSS for Windows version 27.0, licensed to Khon Kaen University (SPSS Inc., Chicago, Illinois, United States).

## 3 Results

During the study period, 1,356 patients diagnosed with sepsis, as indicated by a qSOFA score of ≥2, were admitted to the emergency department. A total of 1,172 patients were excluded for the following reasons: 325 were transferred from other hospitals, 234 were transferred to another hospital, 131 had a history of heart failure, 152 had end-stage chronic kidney disease, 78 had active cancer, 87 had interstitial lung disease, 48 had acute myocardial infarction, 32 had cardiogenic shock, and 85 lacked ultrasound documentation.

Ultimately, 184 patients were enrolled in this study, with a mean age of 63.50 years, of whom 51.08% were men. Most patients were classified as being at triage level 2 (64.67%). The most common etiology of sepsis was pneumonia (84, 45.65%), followed by abdominal sepsis (52, 28.26%) and urosepsis (42, 22.82%). Patient characteristics are summarized in Table 1.

**Table 1:** Patient demographic information (*N* = 184).

Variables	B-lines (*n* = 56)	No B-lines (*n* = 128)	*P* value
Age (year), median (IQR)	64 (51–83)	63 (42–73)	0.325
Men, %	29 (51.78)	65 (50.78)	0.314
Triage level, %			0.345
Level 1	4 (7.14)	11 (8.59)	
Level 2	36 (64.29)	83 (64.84)	
Level 3	16 (28.57)	34 (26.57)	
PaO2/FiO2 ratios, median (IQR)	195 (110–252)	265 (220–455)	0.046
Past medical history, %			0.168
Hypertension	38 (67.85)	89 (69.53)	
Coronary artery disease	5 (19.23)	14 (10.93)	
Asthma/COPD	13 (23.21)	23 (17.96)	
Diabetes	17 (30.35)	53 (41.40)	
Stroke	9 (28.57)	15 (11.72)	
Dementia	13 (23.21)	16 (12.5)	
AIDS	0 (0)	3 (2.34)	
Etiology of sepsis, %			0.247
Pneumonia	26 (46.43)	58 (45.31)	
Intraabdominal infection	17 (30.36)	35 (27.34)	
Urosepsis	13 (23.21)	29 (22.66)	
Soft tissue and MSK infection	0 (0)	6 (4.69)	
Fluid balance at admission (ml), median (IQR)	1,205 (850–2,250)	1,650 (875–3,650)	0.624
Diuretic used	32 (57.14)	44 (34.37)	0.015
Renal replacement therapy	7 (12.5)	9 (7.03)	0.043
Vasopressor used	33 (58.9)	37 (28.91)	0.003

B-lines were detected in 30.43% of patients (*n* = 56), indicating the presence of pulmonary pathology with increased extravascular lung water (OR 4.2, 95% CI 1.1–15.8, *p* = 0.032).

In terms of clinical outcomes, in-hospital mortality was 16.07% (*n* = 9) in the B-lines group and 4.68% (*n* = 6) in the non-B-lines group (*p* < 0.001). Mechanical ventilation was required in 39.28% of patients in the B-lines group, compared to 7.03% in the non-B-lines group (*p* < 0.001). The median hospital length of stay was 11 days for patients with B-lines and 6 days for those without B-lines (*p* < 0.001). This was described in Table 2.

**Table 2:** Clinical outcomes of the study population.

Variables	B-lines (*n* = 56)	No B-lines (*n* = 128)	*P* value
Death at hospital, %	9 (16.07)	6 (4.68)	<0.001
Mechanical ventilation usage, %	22 (39.28)	9 (7.03)	<0.001
Length of mechanical ventilation used (days), median (IQR)	7 (5–12)	4 (1–6)	0.023
Length of hospital stay (days), median (IQR)	11 (5–18)	6 (4–14)	<0.001

In the multivariable logistic regression analysis, the B-lines group exhibited a significantly higher hospital mortality rate (adjusted odds ratio, 4.6; 95% confidence interval, 2.5–5.5) compared to the non-B-lines group. The B-lines group exhibited a significantly higher rate of mechanical ventilation utilization compared to the non-B-lines group (adjusted odds ratio, 9.1; 95% confidence interval, 6.4–11.2) (Table 3).

**Table 3:** Univariate and multivariate analysis according to presence of B-lines in lung ultrasound on clinical outcome.

Variables	Crude odds ratio (95% CI, *P* value)	Adjusted odds ratio (95% CI, *P* value)
Death at hospital	3.89 (1.31–11.53, 0.014)	4.6 (2.5–5.5, 0.021)
Mechanical ventilation usage	8.56 (3.61–20.27, <0.001)	9.1 (6.4–11.2, 0.034)

## 4 Discussion

B-lines are distinct ultrasound artifacts in lung ultrasound, originating at the pleural line, appearing as light ray-like projections that obscure background artifacts, and moving synchronously with lung sliding. The presence of B-lines indicates extravascular lung water, which is associated with various pathologies (17–24).

This study demonstrates that the presence of B-lines detected on LUS is strongly associated with adverse outcomes in adult septic patients presenting to the emergency department. Patients with B-lines had significantly higher rates of in-hospital mortality, mechanical ventilation requirement, ICU admission, and prolonged hospitalization compared to those without B-lines. These findings suggest that LUS can serve as a valuable bedside risk stratification tool in resource-limited emergency department settings, identifying septic patients who warrant closer monitoring and more aggressive supportive care.

The mortality rate in the B-lines group was 16.07%, compared to 4.68% in the non-B-lines group, suggesting that the presence of B-lines may be associated with an increased mortality risk in sepsis patients. Our findings are consistent with prior literature (25–27) demonstrating associations between lung ultrasound findings and adverse outcomes in critically ill patients. However, most previous studies have been conducted in well-resourced settings with access to comprehensive diagnostic modalities.

A significantly higher proportion of patients in the B-lines group required mechanical ventilation (39.28%) compared to the non-B-lines group (7.03%), indicating that B-lines are associated with greater respiratory impairment. Our study also found that the duration of mechanical ventilation was significantly longer in the B-lines group (median of 7 days) compared to the non-B-lines group (median of 4 days). The strong association between B-lines and mechanical ventilation requirement (OR 9.1, 95% CI 6.4–11.2) suggests that B-lines may identify patients with evolving respiratory failure who could benefit from earlier respiratory support interventions, though our study design cannot establish causation (28, 29).

Importantly, our study population had pneumonia as the most common source of sepsis (46.43% in B-lines group vs. 45.31% in non-B-lines group, *p* = 0.247). This underscores that B-lines are nonspecific findings that cannot distinguish between various causes of increased extravascular lung water. Nevertheless, our data demonstrate that B-line presence carries prognostic significance regardless of etiology.

The median hospital stay was significantly longer in the B-lines group (11 days) compared to the non-B-lines group (6 days), reinforcing the association between B-lines and increased illness severity, as well as prolonged hospitalization. This finding is consistent with previous studies indicating extended hospital stays in sepsis-related ARDS. Additionally, some studies have reported prolonged intensive care unit stays for these patients (29, 30).

The clinical importance of these findings pertains to the prognostic, rather than diagnostic, significance of B-lines in septic patients. B-lines are vertical hyperechoic artifacts that signify elevated extravascular lung water, arising from various causes such as pneumonia (the predominant cause of sepsis in our cohort at 46%), cardiogenic pulmonary edema, iatrogenic fluid overload due to resuscitation, chronic interstitial lung disease, or acute respiratory distress syndrome. Our study indicates that, independent of the underlying etiology, the presence of B-lines represents a high-risk cohort with significantly worse outcomes.

Our study confirms that lung ultrasound can serve as a valuable bedside tool for emergency physicians managing critically ill patients, particularly in resource-limited settings where advanced imaging modalities are unavailable. This aligns with studies on the utility of lung ultrasound in diagnosing and managing COVID-19, which suggested that lung POCUS could be a viable alternative to chest CT scans (31–33). Furthermore, our findings highlight the importance of identifying B-lines in the emergency department for patients with sepsis-related ARDS, as it influences clinical outcomes and is crucial for timely resuscitation and intensive care planning (34–36).

This study presents several significant strengths, including its implementation in a resource-limited emergency department setting where point-of-care ultrasound may exhibit optimal clinical utility and impact, the implementation of a standardized lung ultrasound protocol with clear, reproducible criteria for B-line positivity (≥3 B-lines in ≥2 bilateral lung zones), assessment of various clinically relevant results such as in-hospital mortality, necessity for mechanical ventilation, and ICU admission, as well as a sufficient sample size to identify significant associations with substantial effect sizes across multiple endpoints. However, several significant limitations deserve attention. Selection bias presents a significant issue since POCUS was conducted at the discretion of the treating physician instead of following a systematic protocol, potentially biasing our cohort toward more severely ill patients or those with heightened clinical suspicion for pulmonary pathology, thus restricting generalizability for a broader sepsis populations. The temporal ambiguity concerning the timing of ultrasound examinations in relation to fluid resuscitation constitutes a notable limitation; although POCUS was consistently conducted within 2 h of emergency department arrival, we were unable to systematically record the exact timing or volume of fluids administered prior, hindering our ability to differentiate B-lines indicative of pre-existing pulmonary pathology or early sepsis-induced lung injury from those caused by iatrogenic fluid overload. We are unable to define the specific cause of B-lines in individual patients unless they are due to pneumonia, sepsis-induced ARDS, cardiogenic pulmonary edema, or chronic interstitial illness. However, this nonspecificity does not inherently reduce prognostic value, as our findings indicate that B-line detection identifies high-risk patients irrespective of etiology, which may prove more immediately beneficial than an accurate etiological diagnosis in resource-constrained environments. Further methodological limitations encompass the single-center, retrospective design characterized by substantial exclusions, a modest sample size with a limited number of events, leading to low events-per-variable ratios beneath the standard threshold of 10, as well as numerous data deficiencies, including the absence of systematic comparisons with chest imaging, lack of microbiological data, undocumented ventilatory parameters, imprecise timing of interventions (diuretics, renal replacement therapy, vasopressors), and the absence of formal interobserver agreement assessments for B-line interpretation. These constraints highlight the necessity for validation via extensive, prospective, multicenter studies utilizing systematic ultrasound protocols with standardized timing in relation to resuscitation, thorough data collection, and blinded outcome evaluation to corroborate and expand our initial findings.

## 5 Conclusion

Our study indicates that B-lines identified on lung ultrasound are significantly correlated with adverse outcomes in adult septic patients arriving at the emergency department in a resource-constrained environment. B-lines are nonspecific indicators that may arise from various causes such as pneumonia, fluid overload, cardiogenic edema, or ARDS; their presence signifies high-risk patients who face markedly elevated rates of mortality, mechanical ventilation, ICU admission, and extended hospitalization. LUS serves as an efficient, swift, and non-invasive instrument for bedside risk assessment in the absence of advanced diagnostic resources. These findings argue for the incorporation of lung ultrasound in the preliminary evaluation of septic patients to inform clinical decision-making and resource distribution.

## Data Availability

The data analyzed in this study is subject to the following licenses/restrictions: the raw data supporting the conclusions of this article will be made available by the authors, without undue reservation. Requests to access these datasets should be directed to Korakot Apiratwarakul, korakot@kku.ac.th.
